# KINLESSNESS AT THE TIME OF DEATH: IMPLICATIONS FOR END-OF-LIFE DECISION-MAKING

**DOI:** 10.1093/geroni/igz038.885

**Published:** 2019-11-08

**Authors:** Katherine Ornstein, Christine Mair, Marie Skov Kristensen, Melissa Aldridge, Lau Caspar Thygesen

**Affiliations:** 1 Icahn School of Medicine at Mount Sinai, New York, New York, United States; 2 University of Maryland, Baltimore County (UMBC), Baltimore, Maryland, United States; 3 National Institute of Public Health, University of Southern Denmark, København, Hovedstaden, Denmark

## Abstract

As our society continues to age and family size decreases, there is increasing concern about lack of caregiver availability. This may be especially important in the context of end-of-life decision-making. The goal of this study was to characterize the size and composition of the family network of adults at the time of death using a population-based register study. All adults in Denmark who died of natural causes 2009-2016 (n= 401,000) were linked to living adult family members (parents, children, spouses, sibling, great/grandchildren). While the majority of decedents were linked to multiple family members (median =5), 21.6% had no identified living family at the time of their death. Kinlessness was especially common among older women and those with dementia. In addition to supporting caregiving families at the end-of-life, we must also recognize that there are many kinless individuals who may benefit from early formal care planning services to facilitate end-of-life decision-making.

